# Evaluation of Microbe-Driven Soil Organic Matter Quantity and Quality by Thermodynamic Theory

**DOI:** 10.1128/mBio.03252-20

**Published:** 2021-02-23

**Authors:** Jianwei Zhang, Youzhi Feng, Meng Wu, Ruirui Chen, Zhongpei Li, Xiangui Lin, Yongguan Zhu, Manuel Delgado-Baquerizo

**Affiliations:** a State Key Laboratory of Soil and Sustainable Agriculture, Institute of Soil Science, Chinese Academy of Sciences, Nanjing, China; b State Key Laboratory of Urban and Regional Ecology, Research Center for Eco-Environmental Sciences, Chinese Academy of Sciences, Beijing, China; c Key Laboratory of Urban Environment and Health, Institute of Urban Environment, Chinese Academy of Sciences, Xiamen, China; d Departamento de Sistemas Fisicos, Quimicos y Naturales, Universidad Pablo de Olavide, Seville, Spain; e Hawkesbury Institute for the Environment, Western Sydney University, Penrith, New South Wales, Australia; Lawrence Berkeley National Laboratory; Corporación CorpoGen

**Keywords:** thermodynamic quality, bacterial community, soil organic matter, Fourier transform ion cyclotron resonance mass spectrometry, Gibbs free energy, thermodynamic quality

## Abstract

Microbial communities, coupled with substrate quality and availability, regulate the stock (formation versus mineralization) of soil organic matter (SOM) in terrestrial ecosystems. However, our understanding of how soil microbes interact with contrasting substrates influencing SOM quantity and quality is still very superficial. Here, we used thermodynamic theory principles and Fourier transform ion cyclotron resonance mass spectrometry (FTICR-MS) to evaluate the linkages between dissolved organic matter (DOM [organic substrates in soil that are readily available]), thermodynamic quality, and microbial communities. We investigated soils from subtropical paddy ecosystems across a 1,000-km gradient and comprising contrasting levels of SOM content and nutrient availability. Our region-scale study suggested that soils with a larger abundance of readily accessible resources (i.e., lower Gibbs free energy) supported higher levels of microbial diversity and higher SOM content. We further advocated a novel phylotype-level microbial classification based on their associations with OM quantities and qualities and identified two contrasting clusters of bacterial taxa: phylotypes that are highly positively correlated with thermodynamically favorable DOM and larger SOM content versus those which are associated with less-favorable DOM and lower SOM content. Both groups are expected to play critical roles in regulating SOM contents in the soil. By identifying the associations between microbial phylotypes of different life strategies and OM qualities and quantities, our study indicates that thermodynamic theory can act as a proxy for the relationship between OM and soil microbial communities and should be considered in models of soil organic matter preservation.

## INTRODUCTION

Soil organic matter (SOM) is an important regulator of the climate, fertility, and productivity of earth (1, 2). Microorganisms are entwined with SOM cycling (3), as the main drivers of SOM accumulation by controlling the balance between the formation and mineralization processes (4–6). However, despite the importance of these soil microbes and processes, the mechanisms behind these microbe-SOM associations remain poorly understood. This is, in part, a consequence of the immense biodiversity of microbes thriving in our soils, which are often associated with complex metabolic substrates (7, 8). In addition to the inherent chemical heterogeneity, substrate bioavailability was also influenced by many factors: for example, charged minerals can bind even easily degradable substrates and make them inaccessible to microbes (9). Aiming to gain a deeper understanding of the microbe-OM associations, ecologists have tried to classify microbial taxa based on their life strategies and resource preferences (e.g., copiotrophs versus oligotrophs [9–11]). However, very little progress has been made on this endeavor over the past decade (9–11). Due to these distinct life strategies, copiotrophic and oligotrophic microbes are expected to play contrasting roles in regulating soil carbon cycling (9, 12, 13). In theory, copiotrophs are fast growers with low substrate use efficiency, while oligotrophs grow efficiently at lower growth rates and have a competitive advantage under resource-limited conditions (8, 10). However, despite the theoretical advances of knowledge (14–16), we still lack a solid comprehensive classification for microbial phylotypes based on their utilization of organic matter substrates with different levels of quality and bioavailability and different thermodynamic properties.

Classification of microbial phylotypes based on their substrate preferences is essential to advance our understanding on how microbial communities associated with SOM accumulation (17). Herein we argue that the principles of thermodynamic theory applied to dissolved organic matter (DOM) could be used to advance our understanding of the mechanisms by which microbial phylotypes contribute to SOM formation and mineralization. Observational evidence has indicated that DOM quality can shift the composition of the microbial community as a whole and the proportion of phylotypes (18). Moreover, we know that the degree of oxidation and the quality of DOM substrate (i.e., thermal breakdown of DOM) regulate the capacity of soil microbes to transform and mineralize more recalcitrant SOM (9, 12). For instance, substrates with lower thermodynamic qualities (i.e., a less thermodynamically favorable substrate) are expected to provide limited resources for microbes (19, 20) and, therefore, might reduce the diversity of soil organisms feeding on these substrates (18). Moreover, less favorable substrates, which are often reported to be associated with a lower soil carbon stock and poor nutrient resource status (21, 22), might be associated with very specific microbial phylotypes capable of thriving in poor-quality substrates. Therefore, elucidation of the associations between microbial taxa and DOM quality with regard to its thermodynamic properties is fundamental to providing a new mechanistic hypothesis on how microbial communities regulate the formation of C stocks through SOM accumulation and mineralization processes.

To advance our understanding of the mechanisms of microbial-SOM association with regard to SOM thermodynamic properties at the molecular level, we sampled a wide gradient of soil types with contrasting nutrient statuses and SOM contents from six field experimental sites established in paddies across subtropical China (23). Paddy soils, with nearly 150 million hectares under cultivation globally (24), play a key role in feeding an ever-increasing global population and are essential to climate regulation and global C stocks (25). To address this important knowledge gap, we used electrospray ionization coupled with the Fourier transform ion cyclotron resonance mass spectrometry method to obtain site-scale information on the DOM thermodynamic quality at the molecular level. Instead of directly quantifying the exact energy state, this approach has been proven to be a feasible method for estimating the energetic potential (e.g., Gibbs free energy [Δ*G*°]) of naturally occurring organic compounds based on their chemical composition (i.e., the ratio of major elements C, H, N, O, S, and P) (26). A higher Gibbs free energy for a given organic compound indicates reduced thermodynamic quality and vice versa (20). Thus, the application of Gibbs free energy was expected to overcome the shortcomings of the diversified substrates and substrate preferences of microbial taxa. Our study aims to advance our mechanistic knowledge of the associations between substrate thermodynamic quality and microbe-driven carbon cycling and advance our ability to predict soil carbon dynamics as well as their corresponding ecological implications.

## RESULTS

### Characterizing microbial taxa and thermodynamic OM qualities across contrasting edaphic conditions.

**FIG 1:**
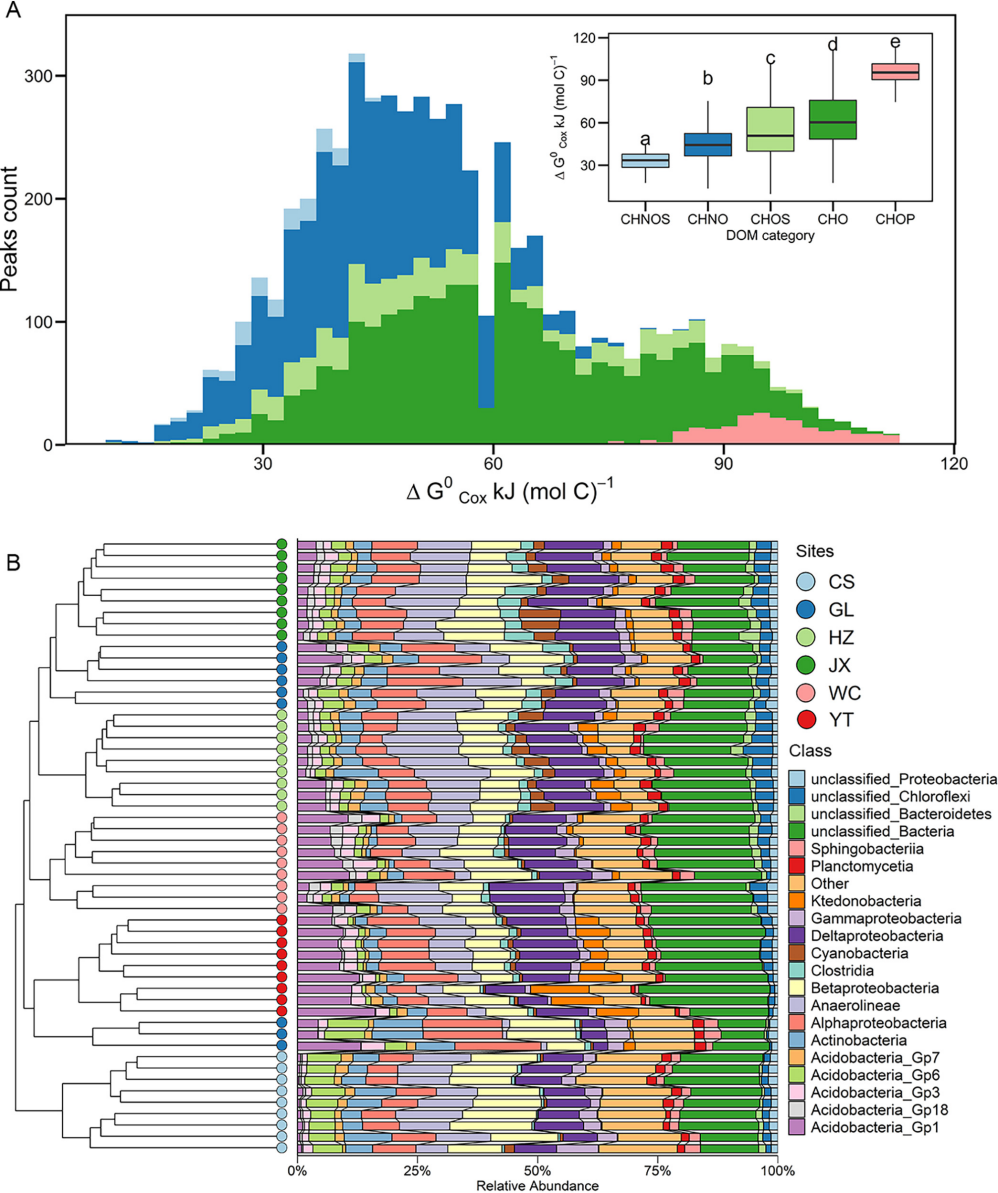
Compositions of DOM molecular and bacterial community across sampling sites. (A) Distributions of DOM molecules (*n* = 5,254) of 54 paddy soils across six sites (main plot). Different colors denote their distinct chemical compositions. Differences in molecular $\Delta G_{\text{Cox}}^{\circ }$ among DOM categories were evaluated with one-way ANOVA followed by *post hoc* Tukey’s HSD tests, and different letters indicate significant differences (subplot). (B) Cluster analysis showed the compositional difference of bacterial community around sampling sites at the genus and class levels.

Our study system provides wide gradients of DOM qualities and diversity and relative abundances of bacterial phylotypes. We identified 21,253 DOM compounds, with molecular weights ranging from 156 to 754.2 Da, within which 9,295 have formula assignments (see Fig. S1 in the supplemental material). Our results showed great heterogeneity of DOM chemical composition across sampling sites. For example, lipids were more enriched in JX and YT, tannins were more enriched in CS and GL, and condensed aromatics were more enriched in CS and HZ (see Fig. S2 in the supplemental material). To eliminate the interference of those sparse mass spectrometry (MS) peaks, we further refined our raw MS data into a dominant subset for further analysis, which still depicted the biochemical composition of overall MS data with high level of confidence (Fig. S1). The dominant DOM molecules in the sampled paddy soil mainly belong to the CHO (44.54%) and CHNO (36.90%) classes, followed by CHOS (12%) (Fig. 1A), which presents a wide range of DOM thermodynamic qualities. Phosphonate compounds consistently had the highest Gibbs free energy for the half-reaction of carbon oxidation $\left(\Delta G_{\text{Cox}}^{\circ }\right)$ value, despite their low presence (3.86%) in the DOM molecular profiles (Fig. 1A). Nitrogenous compounds consistently exhibit higher $\Delta G_{\text{Cox}}^{\circ }$ values than N-free compounds (Fig. 1A). For site heterogeneity, the DOM median $\Delta G_{\text{Cox}}^{\circ }$ among the samples was consistently higher in YT than those in CS, regardless of fertilization regimen (see Fig. S3 in the supplemental material).

10.1128/mBio.03252-20.3 FIG S1 Validation of condensed DOM data set used in the manuscript compared with the raw data set. (a) Frequency of DOM compounds detected in our MS profiles, which was extremely skewed. In order to eliminate the interference of those sparse DOM compounds, we selected a subset containing DOM compounds detected more than six times for further analysis. (We have six sampling sites in this study.) (b) Distribution of MS peaks per sample in raw data and condensed (USED) data. (c) Relationship between biochemical composition (dissimilarity) based on Bray-Curtis distance for the condensed subset used in this study (5,254 compounds) and the raw data (21,253 compounds). (d) The relationship between median Gibbs free energy per sample for the condensed subset used in this study and the raw data suggests the strongly positive correlation in the same scale. Download FIG S1, JPG file, 1.2 MB. Copyright © 2021 Zhang et al. 2021 Zhang et al. https://creativecommons.org/licenses/by/4.0/ This content is distributed under the terms of the Creative Commons Attribution 4.0 International license.

10.1128/mBio.03252-20.4 FIG S2 Kernel density plots of DOM formula in van Krevelen diagrams (raw MS data). Darker color indicates a higher molecular density. Rectangular areas denote the assigned molecular category and corresponding count ratio. Filled and blank dots represent DOM compounds that could and could not be assigned to a specific molecular category, respectively. Download FIG S2, JPG file, 2.2 MB. Copyright © 2021 Zhang et al. 2021 Zhang et al. https://creativecommons.org/licenses/by/4.0/ This content is distributed under the terms of the Creative Commons Attribution 4.0 International license.

10.1128/mBio.03252-20.5FIG S3DOM median Gibbs free energy $\left(\Delta G_{\text{Cox}}^{\circ }\right)$ under different fertilization regimens across six paddy soils. CK, no fertilization; NPK, chemical fertilization; OM, organic fertilization. Within each site, OM treatment delivered soil of higher fertility than NPK and unfertilized treatments. Significant differences among sampling sites within each fertilization treatment by one-way ANOVA are labeled above boxes with asterisks: *, *P* < 0.05; **, *P* < 0.01; and ****, *P* < 0.0001. Different letters above box plots denote significant differences among sample pairs (*post hoc* Tukey’s test). Download FIG S3, JPG file, 0.6 MB.Copyright © 2021 Zhang et al.2021Zhang et al.https://creativecommons.org/licenses/by/4.0/This content is distributed under the terms of the Creative Commons Attribution 4.0 International license.

The bacterial community also significantly varied among the samples: *Actinobacteria* (1.06%∼11.80%), *Acidobacteria*_Gp1 (0.31%∼16.14%), *Anaerolineae* (0.87%∼16.81%), and *Alpha*- (4.47%∼11.94%), *Beta*- (6.01%∼17.09%), and Deltaproteobacteria (3.04%∼15.69%) were the dominant bacterial lineages at the class level (Fig. 1B). Additionally, *Alpha*-, *Beta*-, and *Gammaproteobacteria* and *Anaerolineae* were consistently rare in YT samples (Fig. 1B; see Fig. S4 in the supplemental material). *Acidobacteria*_Gp1 and *Ktedonobacteria* were enriched in YT but were relatively rare in CS samples. Moreover, *Acidobacteria*_Gp1 and *Acidobacteria*_Gp3 were enriched in WC and YT, but members of *Acidobacteria*_Gp6 were relatively rare at these two sites (Fig. S4).

10.1128/mBio.03252-20.6 FIG S4 Box plots showing regional differences of top classes. Significant differences among sampling sites by one-way ANOVA are labeled above boxes with asterisks: *, *P* < 0.05; **, *P* < 0.01; ***, *P* < 0.001; and ****, *P* < 0.0001. Different letters above box plots denote significant differences among sample pairs (*post hoc* Tukey’s test). Download FIG S4, JPG file, 1.8 MB. Copyright © 2021 Zhang et al. 2021 Zhang et al. https://creativecommons.org/licenses/by/4.0/ This content is distributed under the terms of the Creative Commons Attribution 4.0 International license.

### Linking DOM thermodynamic quality to microbial diversity and community composition.

**FIG 2:**
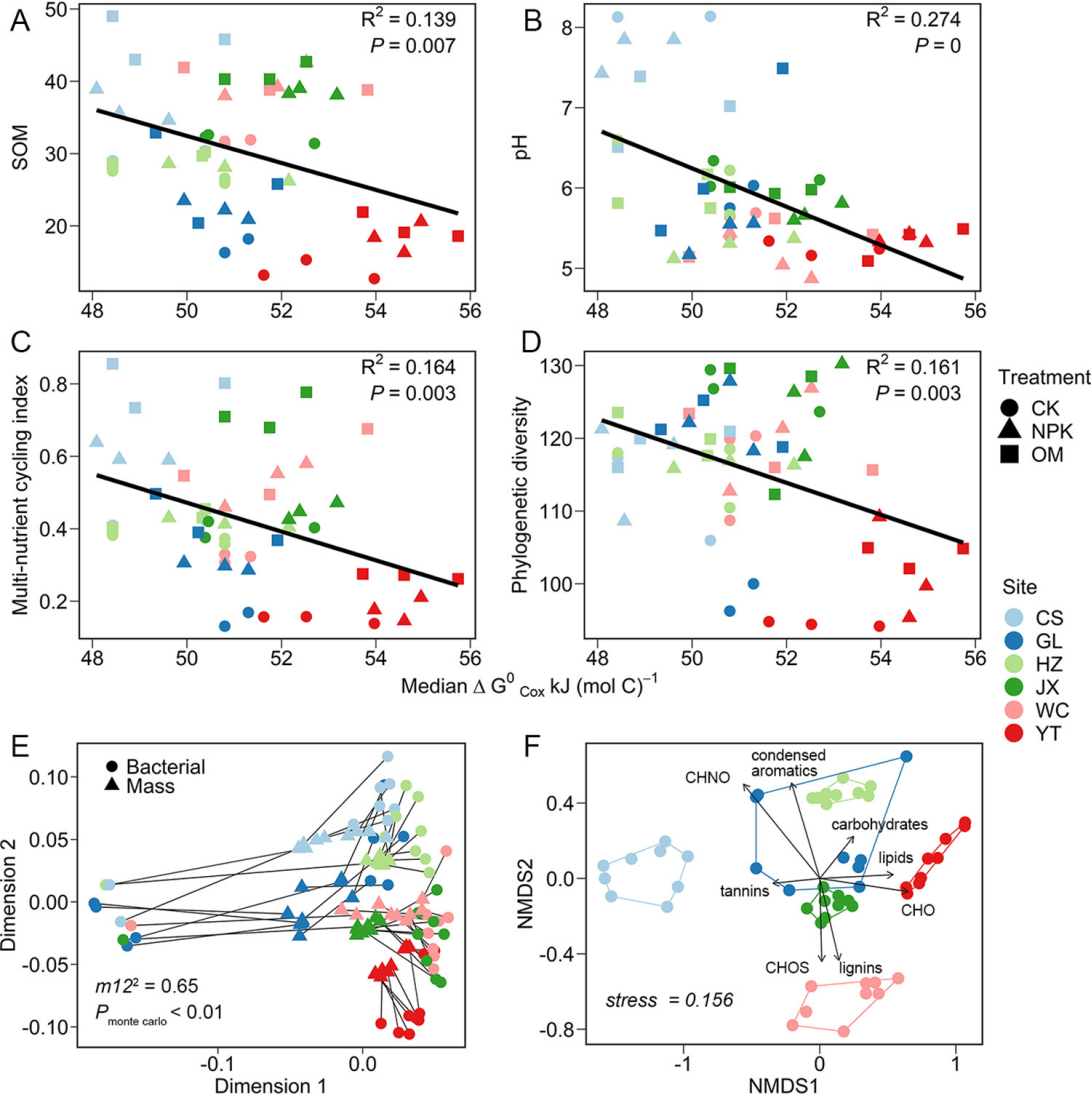
Relationships between DOM thermodynamic quality and SOM content (A), soil pH (B), and soil multinutrient cycling (C), as well as bacterial phylogenetic diversity (D). The significant correlation between DOM molecular composition and bacterial community was determined by Procrustes analysis (E). Multivariate analysis of bacterial community and drivers using nonmetric multidimensional scaling (NMDS) based on Bray-Curtis dissimilarity (F). DOM factors community were fit to the ordination using the envfit function, respectively. Only factors with a significance level of *P* < 0.05 are shown.

We found strong associations between the DOM thermodynamic qualities and microbial community composition and diversity as well as SOM contents (Fig. 2 [and see Fig. 4 below]; Fig. S3). We showed that increasing DOM median $\Delta G_{\text{Cox}}^{\circ }$ was significantly correlated with lower SOM content (*R*^2^ = 0.139, *P* < 0.01 [Fig. 2A]) and lower soil pH (*R*^2^ = 0.274, *P* < 0.01 [Fig. 2B]). This result also coincides with the change in the soil multifunctionality (*R*^2^ = 0.149, *P* < 0.01 [Fig. 2C]). We also found negative relationships between the DOM median $\Delta G_{\text{Cox}}^{\circ }$ and bacterial diversities, with significance at the phylogenetic level (*R*^2^ = 0.161, *P* < 0.01 [Fig. 2D]) and marginal significance at the taxonomic level (*R*^2^ = 0.114, *P* = 0.051 [see Fig. S5 in the supplemental material]), and these correlations were not biased with regard to sampling effort (Fig. S5). Moreover, the Mantel test revealed that the bacterial taxonomic and DOM biochemical compositions were significantly correlated (Mantel *r* = 0.2284, *P* < 0.01), which was also confirmed by the Procrustes analysis (*m12*^2^ = 0.69, *P*_Monte Carlo_ < 0.01 [Fig. 2E]). To confirm these findings, we fitted DOM factors to unconstrained nonmetric multidimensional scaling (NMDS) ordination of the bacterial communities (Bray-Curtis distance) (Fig. 2F). Significant correlations (*P* < 0.05) were observed between the variances of bacterial composition and CHNO (*R*^2^ = 0.564), CHO (*R*^2^ = 0.428), CHOS (*R*^2^ = 0.194), condensed aromatics (*R*^2^ = 0.300), lipids (*R*^2^ = 0.292), lignins (*R*^2^ = 0.206), and tannins (*R*^2^ = 0.119) (Fig. 2F).

10.1128/mBio.03252-20.7FIG S5(a) The consistency of bacterial taxonomic richness and phylogenetic diversity driven from rarefied and nonrarefied community data sets. (b) Correlation between bacterial diversity and DOM thermodynamic quality (DOM median Gibbs free energy $\left[\Delta G_{\text{Cox}}^{\circ }]\right)$ are not biased by sampling effort. Download FIG S5, JPG file, 1.0 MB.Copyright © 2021 Zhang et al.2021Zhang et al.https://creativecommons.org/licenses/by/4.0/This content is distributed under the terms of the Creative Commons Attribution 4.0 International license.

### Classification of the dominant bacterial phylotypes based on their diverging associations with the thermodynamic quality of the DOM and SOM content.

**FIG 3:**
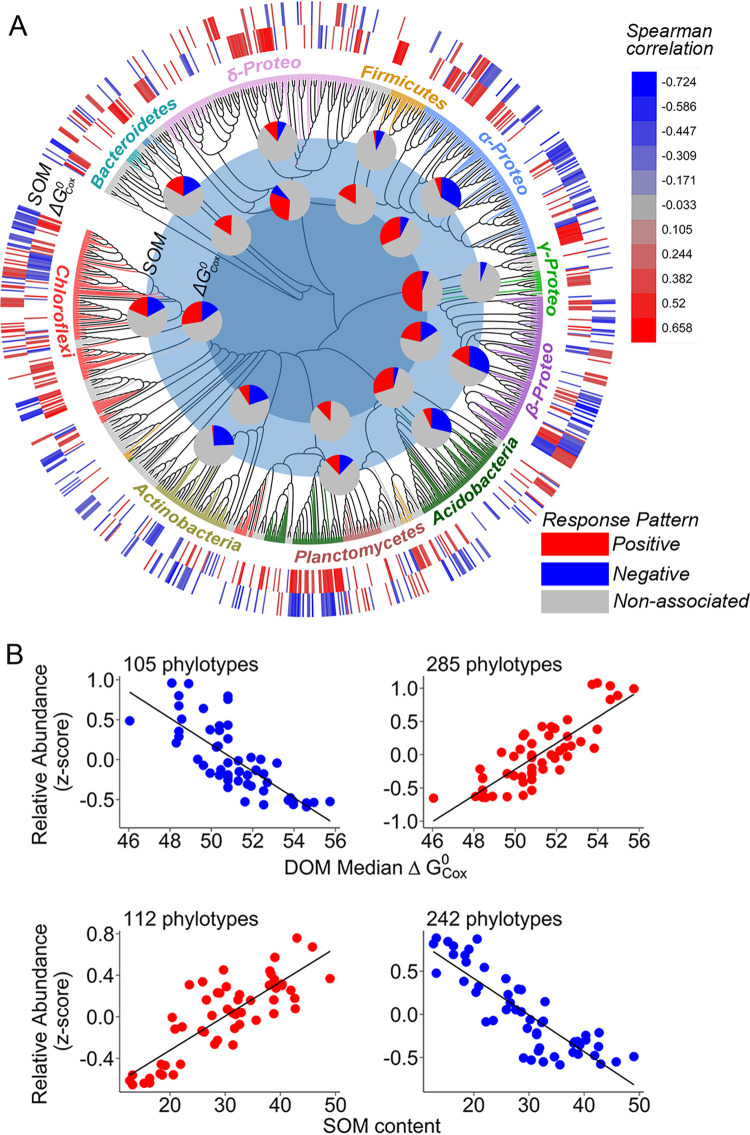
Identified OM preference for dominant soil bacterial phylotypes and potential implications for SOM stock. (A) Phylogenetic distribution of the 1,152 dominant bacterial phylotypes with branches colored by taxonomic assignment and their Spearman correlation coefficients with the DOM median $\Delta G_{\text{Cox}}^{\circ }$ (inner wide ring) and SOM content (outer wide ring). Pie charts show the distribution of response patterns within each dominant phylum to DOM median $\Delta G_{\text{Cox}}^{\circ }$ (inner pie charts) and their potential implication for SOM content (outer pie charts). A nonassociated phylotype (gray in pie charts and blank in wide rings) denotes no significant correlation at the 0.05 level. (B) Relationships between the averaged relative abundance of the phylotypes positively and/or negatively correlated with DOM median $\Delta G_{\text{Cox}}^{\circ }$ as well as SOM content.

**FIG 4:**
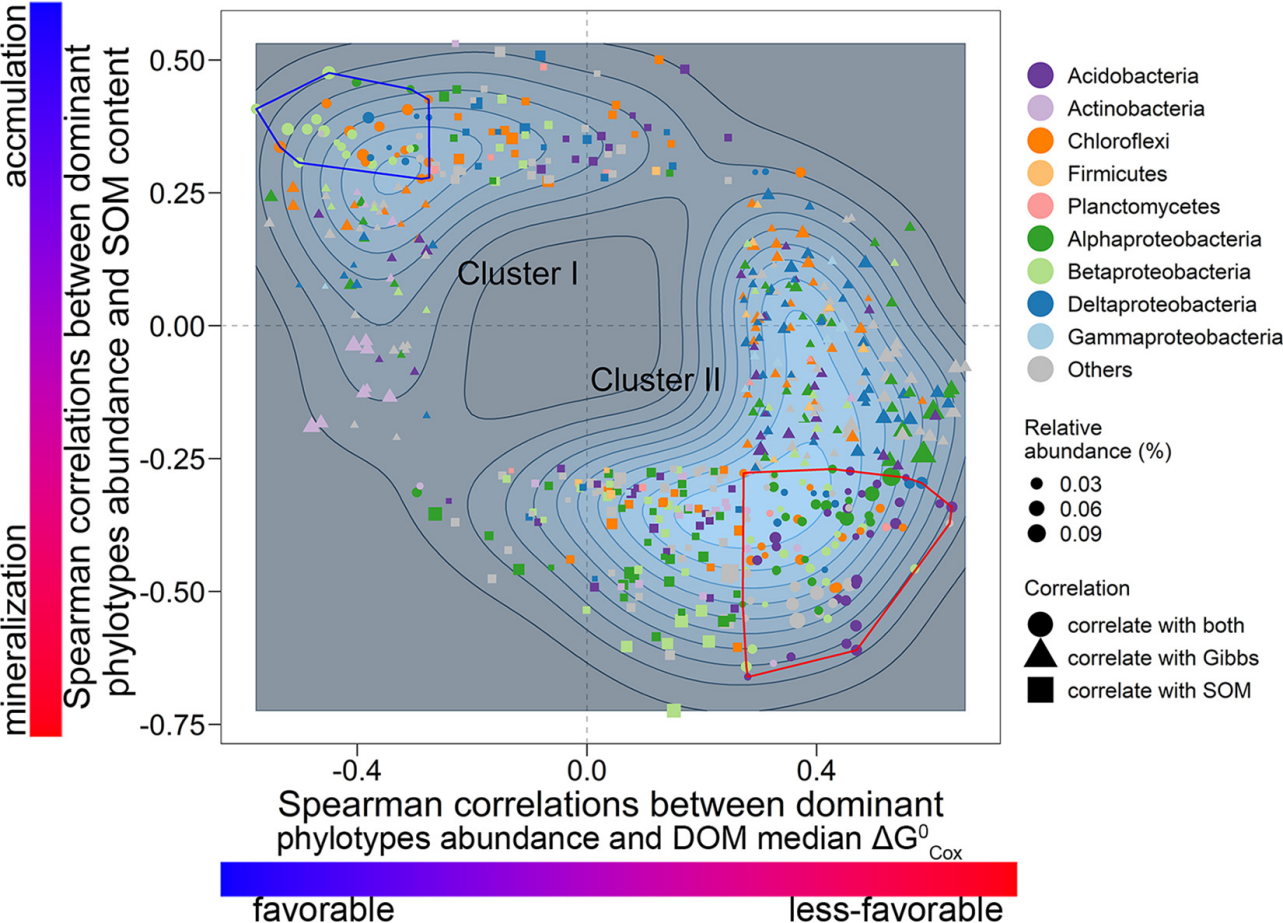
Classification of dominant bacterial phylotypes (*n* = 593) based on their significant Pearson correlation (*P* < 0.05) with DOM median $\Delta G_{\text{Cox}}^{\circ }$ and/or SOM content. Dominant phylotypes that were not significantly correlated with either DOM median $\Delta G_{\text{Cox}}^{\circ }$ or SOM content are not shown. Bacterial phylotypes were mainly classified into two clusters. Members in red and blue polygons were significantly correlated with both DOM median $\Delta G_{\text{Cox}}^{\circ }$ and SOM content.

To further investigate the linkages between microbial phylotypes with DOM quality and SOM content at a finer level of resolution, we focused on 1,152 ubiquitous phylotypes that were present in more than half of our experimental sites (>4/6 sites [Fig. 3]). We then classified the dominant bacterial phylotypes based on their shared preference for organic substrate quality and potential implications for SOM content, restricting our analysis to those 593 phylotypes that were significantly (*P* < 0.05) correlated with DOM thermodynamic quality and/or SOM content. The dominant phylotypes were classified into two clusters without clear boundaries, with cluster I containing fewer members than cluster II (Fig. 4; see Table S2 in the supplemental material). Specifically, the members in cluster I were negatively correlated with the DOM median $\Delta G_{\text{Cox}}^{\circ }$ and positively correlated with the SOM content (Fig. 4). Of those taxa in cluster I, 39 phylotypes, such as the genus *Thiobacillus* in *Betaproteobacteria*, were significantly correlated with both the DOM median $\Delta G_{\text{Cox}}^{\circ }$ and SOM content (blue polygon). In contrast, cluster II consisted of bacterial phylotypes that were positively correlated with the DOM median $\Delta G_{\text{Cox}}^{\circ }$ and/or negatively correlated with the SOM content (Fig. 4); 107 phylotypes were significantly correlated with both DOM median $\Delta G_{\text{Cox}}^{\circ }$ and SOM content (red polygon), such as the genus *Rhizomicrobium* in the *Alphaproteobacteria* and Gp1 to -3 in the *Acidobacteria* (Table S2). Moreover, we found that the associations of the dominant phylotypes with DOM median $\Delta G_{\text{Cox}}^{\circ }$ and SOM content were not phylogenetically clustered.

## DISCUSSION

Integration of microbes in models of global carbon dynamics has gained widespread attention in the past decade (6, 9, 27, 28), and many investigations have reported positive associations between soil microbial diversity and SOM content (18, 29). However, the more specific links between microbial species and carbon cycling are still missing, in part, because of the immense diversity of microbial communities and C substrates in our soils. This knowledge gap limits our capacity to use microbial information in the next generation of soil carbon models. Substrate quality has been reported to be influential in shaping microbial communities and, in turn, soil carbon cycling (8, 13). To date, most relevant documented studies have been conducted in well-controlled microcosms using a specific substrate with discrete chemical (or thermodynamic) quality (such as sucrose, glucose, hemicellulose or cellulose, or lignin) (11–13), which are very different from the natural environmental conditions, wherein substrates consist of more complex molecular compounds (30, 31). Here, we coupled an ultrahigh-resolution mass spectrometry with a large-scale soil survey, including contrasting soil properties (i.e., soil pH and SOM content) and microbial communities (microbial composition and diversity). This allowed us to obtain a high-resolution identification of the heterogeneity in molecular biochemical profiles of naturally occurring organic compounds at a large spatial scale. By doing so, we obtained enough resolution to address our research questions to investigate the links between microbial communities and substrate diversity and composition and to advance our knowledge on the potential microbial mechanisms driving SOM quantity and quality. We reported that thermodynamic theory could help to inform microbe-driven SOM quantity and quality, linking organic substrate qualities and specific microbial species. In this context, soils with organic substrates of higher thermodynamic quality have higher bacterial diversities and in turn more SOM content. Additionally, our study provided a novel species-level classification linking the bacterial community and soil carbon cycling. We acknowledged that beyond the inherent chemical heterogeneity, the bioavailability of substrate is also regulated by others factors, which can outweigh the microbe-substrate interactions. For example, labile components of plant litter could form mineral-stabilized mixtures or even promote aggregation and be protected by spatial inaccessibility (9). Nonetheless, our results advance our understanding of how the microbial community is connected to the chemical environment in ways that can influence importance ecological process, such as SOM cycling in this study.

Biodiversity is crucially important for maintaining ecological functions, and drastic loss in biodiversity could be detrimental to ecosystem stability and human well-being (32, 33). Here, we observed that bacterial diversities were significantly and positively correlated with the thermodynamic quality of the DOM substrate, which exhibited wide gradients at the regional scale (Fig. 2; Fig. S5). This phenomenon is supported by the notion that readily degradable substrates could support diversified microbial communities (18). On the one hand, substrate bioavailability could directly affect microbial metabolism and shift microbial community composition. Specifically, easily degradable substrates could feed all microorganisms with different efficiencies and support a high level of diversity (18, 34). However, recalcitrant substrate is degradable by only a limited number of microbial lineages with specific metabolic capabilities and is thus coupled with lower biodiversity (8, 18). On the other hand, we found that substrate thermodynamic quality could provide combined information about soil abiotic conditions, such as soil pH and ground nutrient status. Specifically, soils with organic substrates of higher thermodynamic quality were observed to be coupled with neutral soil pH and enriched multinutrient cycling (Fig. 2B and C), the latter of which played important roles in shaping diversified niche conditions for microorganisms (e.g., less microbial diversity in acidic and nutrient-limited soils) (35–37). These close associations between substrate thermodynamic quality and other edaphoclimatic factors, which were also evidenced before (38), collectively suggested that thermodynamic information about metabolic substrates in natural ecosystems constitutes an important complement explanation for microbial function and hence SOM accumulation (39).

We also found a positive association between substrate thermodynamic quality and SOM content (Fig. 2). Recently, more attention has been paid to the contribution of microorganisms to SOM accumulation (6, 9, 12). For example, a recent meta-analysis estimated that microbial necromass can make up more than half of the soil organic carbon in agricultural and grassland ecosystems (40). One of the potential mechanisms is that high microbial diversity can build up SOM accumulation (6, 9, 12); however, empirical evidence for this is lacking. Diversified microbial communities assimilate exogenous plant input, synthesizing biomass with different efficiencies, and their necromass is further transformed into a persistent SOM pool, an effect known as the “microbial carbon pump” (9, 12, 41). In this process, substrate quality is the primary controller of SOM accumulation, as it regulates the efficiency of the microbial biomass transformation versus the effect on decomposing SOM (8, 9). Organic substrate serves as sources of both carbon (biomass yielding) and energy (energy yielding) for microorganisms, and microbial growth efficiency stops when the combustion heats of substrate and biomass are equal (42). A thermodynamically favorable substrate is supposed to be correlated with higher CUE (i.e., higher biomass yielding [43]) and hence higher SOM content as a result (44). Specifically, in an environment with an organic substrate of high thermodynamic quality, greater diversity is expected to increase microbial transformation efficiency and consequently generate more microbially derived SOM (10, 21, 22). In this context, microbial diversity allows high OM quality to result in higher organic matter content, connecting OM decomposition and C accumulation in soil. Additionally, the preferential microbial utilization of favorable substrates would protect native recalcitrant SOM from extra microbial degradation (10, 45). In contrast, organic substrates with lower thermodynamic qualities not only limit the microbial transformation of exogenous C into more stable SOM (46) but also facilitate the decomposition of native recalcitrant SOM components for mining nutrients (10, 13). For such reasons, we observed a positive association between the DOM thermodynamic quality and SOM content (Fig. 2A). Moreover, similar outcomes were observed for the majority of any five sites or any four sites (see Fig. S7 in the supplemental material). For example, 15 out of 21 site combinations (71.43%) reported significant associations between thermodynamic quality of DOM and bacterial phylogenetic diversity. In contrast, at the small spatial scales (less than four sites), only 42.86% (15 out of 35) had significant associations. Accordingly, 11 out of 21 site combinations (52.38%) showed significant associations between thermodynamic quality of DOM and SOM content at the large scales, and only 42.86% (15 out of 35) present similar patterns at the small scales. This information indicates that more confidence in this outcome is available for the large scales.

Microbial phylotypes are physiologically diverse, with various substrate transformation efficiencies (8), and are thought to play different roles in regulating soil organic carbon accumulation. We observed that that bacterial community composition was closely correlated with the DOM biochemical composition (Fig. 2E and F), indicating divergent preferences for substrate quality among the bacterial phylotypes. Specifically, we observed that the associations between the bacterial phylotypes and DOM thermodynamic qualities as well as SOM content are not maintained at coarse levels of phylogenetic resolution (e.g., *Betaproteobacteria* and *Chloroflexi*) (Fig. 3 and 4; see Fig. S6 in the supplemental material), indicating that the putative oligotrophic/copiotrophic paradigm could not fully predict the roles of microbial phylotypes in various life strategies in soil carbon cycling (7). This inconsistency could be due to two factors: (i) taxonomic and metabolic diversities of species make it hard to classify their life strategies at the phylum level, and (ii) diversified specific substrates and substrate preferences of microbial taxa make it hard to exactly classify life strategies of whole microbial species in a given community.

10.1128/mBio.03252-20.8FIG S6Relationships between the relative abundances of dominant bacterial lineages at the class level and DOM median Gibbs free energy $\left(\Delta G_{\text{Cox}}^{\circ }\right)$. Linear model expectations are indicated by solid lines. The significant difference was identified at *P* < 0.05. Download FIG S6, JPG file, 3.0 MB.Copyright © 2021 Zhang et al.2021Zhang et al.https://creativecommons.org/licenses/by/4.0/This content is distributed under the terms of the Creative Commons Attribution 4.0 International license.

10.1128/mBio.03252-20.9FIG S7Relationships between the DOM median Gibbs free energy $\left(\Delta G_{\text{Cox}}^{\circ }\right)$ and bacterial phylogenetic diversity as well as SOM content at different spatial scales. Download FIG S7, JPG file, 1.1 MB.Copyright © 2021 Zhang et al.2021Zhang et al.https://creativecommons.org/licenses/by/4.0/This content is distributed under the terms of the Creative Commons Attribution 4.0 International license.

Our study provides a novel species-level classification linking bacterial communities to soil carbon cycling by overcoming the above-mentioned issues. As mentioned above, the application of Gibbs free energy is expected to overcome the shortcomings of diversified specific substrates and substrate preferences of microbial taxa. Moreover, the high resolution used in our study can help identify substrate preference for every taxon, which gives us more comprehensive knowledge of microbe-driven soil carbon cycling. Based on the degree of associations between the bacterial phylotypes and DOM thermodynamic quality as well as SOM content, we identified two contrasting groups of bacterial species, including members from all phyla (Fig. 3 and 4). Cluster I consisted of phylotypes that were positively correlated with the thermodynamic quality of DOM and coupled with larger SOM accumulation. As distinct evidence, *Thiobacillus* in cluster I is a facultative autotrophic bacterium that prefers organic matter with low molecular weights (47, 48). Thus, these phylotypes are more responsive to organic matter upon availability and tend to possess a higher efficiency for biomass transformation (7, 8). In contrast, members of cluster II were consistently associated with less favorable DOM, as well as lower SOM content. These types are characterized by the ability to grow under lower levels of substrate bioavailability (8). For instance, *Rhizomicrobium* tends to be dominant under resource-limited conditions, such as the later phase of straw decomposition (49) or in vegetation restoration after desertification (50). However, their lower growth rate would limit biomass production and SOM transformation efficiency (6). Moreover, we reported that the relative abundances of these dominant phylotypes, both individually and when combined, were significantly correlated with the DOM thermodynamic quality as well as SOM content (Fig. 3).

### Conclusions.

Our findings provide empirical evidence that organic substrate thermodynamic quality plays a critically important role in the regulation of SOM quantity and quality by shifting microbial diversity and community composition at the regional scale. Furthermore, we provide a novel species-level classification linking microbial phylotypes from different lineages with contrasting carbon life strategies. Considering the changes in the chemical composition of the exogenous organic matter input attributed to global change, our study highlights the importance of substrate thermodynamic quality for the maintenance of soil carbon stock. Finally, our work advances our knowledge on the important linkages between microbial taxa and the thermodynamic quality of metabolic substrate to better understand SOM accumulation and manage its preservation.

## MATERIALS AND METHODS

### Field sampling.

Soil samples were collected from six agroecological experimental sites across subtropical China (51). These sites, developed on rice paddy fields, are located across an ∼1,000-km environmental gradient and include different SOM contents, which range from 12.7 to 49 g kg^−1^, and soil pHs, which range from 4.87 to 8.14 (see Table S1 in the supplemental material). All the locations contained three fertilization regimens (control [without fertilization], N-P-K [mineral NPK fertilizers], and OM [mineral fertilizers plus organic amendments]) and three replicates for each treatment. The combination of experimental sites and nutrient treatments (*n* = 54 samples) provided a wide range of nutrient and SOM conditions. To capture the spatial heterogeneity of each location, each soil replicate was a composite sample from 10 cores (top 10 cm). Sampling was conducted in 2014 after the harvest of the paddy rice. After sieving, approximately 10 g and 20 g of each soil sample were stored at −80°C for DNA extraction, and the other subsamples were air dried for analysis of their chemical properties. The SOM content was determined by potassium dichromate oxidation and back titration of excess potassium dichromate using an ammonium ferrous sulfate solution (52).

10.1128/mBio.03252-20.1 TABLE S1 Detailed information on field experimental sites. Download Table S1, XLSX file, 0.01 MB. Copyright © 2021 Zhang et al. 2021 Zhang et al. https://creativecommons.org/licenses/by/4.0/ This content is distributed under the terms of the Creative Commons Attribution 4.0 International license.

10.1128/mBio.03252-20.2TABLE S2Detailed taxonomic information on bacterial phylotypes detected in Spearman correlation with DOM median $\Delta G_{\text{Cox}}^{\circ }$ and SOM content. Download Table S2, XLSX file, 0.07 MB.Copyright © 2021 Zhang et al.2021Zhang et al.https://creativecommons.org/licenses/by/4.0/This content is distributed under the terms of the Creative Commons Attribution 4.0 International license.

### DOM sample preparation using solid-phase extraction.

The DOM was extracted by a solution of 0.5 M K_2_SO_4_ with a liquid/solid ratio of 10:1 (wt/vol) in a reciprocal shaker for 8 h (200 rpm at 25°C). After centrifugation at 5,000 × *g* for 10 min, the suspensions were filtered through a 0.45-μm-pore sterile membrane filter (53). Bulk DOM samples were cleaned by the solid-phase extraction (SPE) process prior to MS analysis. Briefly, the SPE cartridges (Varian Bond Elute PPL, 1 g/6 ml; Agilent Technologies) were rinsed with 2 volumes of methanol (high-performance liquid chromatography [HPLC] grade; Sigma-Aldrich) and 0.01 M HCl immediately before use (30). Equal amounts of each DOM sample (∼100 μg C) were acidified with pure HCl to pH 2 to increase extraction efficiency and passed through the SPE cartridges by gravity (2 ml/min) (31). Then the cartridges were rinsed with two volumes of 0.01 M HCl for the complete removal of salts and then dried using a stream of ultrapure N_2_. DOM was collected by eluting cartridges using methanol (30). The elutes were stored in acid-washed (and combusted) glass vials and kept in the dark in the refrigerator (−20°C) prior to MS measurement.

### FT-ICR MS analysis.

Bulk DOM extractions were characterized by Fourier transform ion cyclotron resonance mass spectrometry (FT-ICR MS) on a Bruker Apex-Ultra mass spectrometer equipped with a 9.4-T actively shielded superconducting magnet interfaced with a negative-ion-mode electrospray ionization system (31). Diluted Suwannee River natural organic matter solution (50 mg liter^−1^) (obtained from IHSS [the International Humic Substances Society]) was used as a control and injected every 18th sample to ensure instrument stability. Deuterated stearic acid (C_18_D_35_H_1_O_2_; Sigma-Aldrich) was loaded as an internal standard to compare the relative intensities of MS spectra in various samples (54). Ammonium hydroxide was added prior to electrospray to increase the ionization efficiency (55). Samples were injected into the electrospray source (3 μl min^−1^, 4.0-kV emitter voltage, 4.5-kV capillary column introduce voltage, and 320-V capillary column end voltage) using the syringe pump (31). Individual scans (a total of 128 with an ion accumulation time of 1 s, an ion flight time into the ICR cell of 1.2 ms, and an *m*/*z* range of 150 to 1,000) were averaged for each sample and internally calibrated using an OM homologous series separated by 14 Da (-CH_2_ group) (20). The data size was set to 4 million words. PPL extraction blanks and solvent blanks were prepared and analyzed to check for contamination and carryover; peaks found in these blanks were removed from the profiles obtained for the DOM samples.

### Molecular formula assignment.

The raw spectral data of the distinct organic compounds (i.e., each unique *m*/*z* peak) with their corresponding intensities were acquired. DataAnalysis software 4.2 (Bruker Daltonik GmbH, Bremen, Germany) was used to convert the raw spectra to a list of *m*/*z* values using FTMS Peak Picker. Detected mass peaks with a signal-to-noise ratio (S/N) of less than 6 were discarded to increase accuracy. To reduce cumulative errors, all the sample peak lists for the entire data set were aligned with each other to eliminate possible mass shifts (20, 45). Putative chemical formulas were assigned using an EMSL in-house software based on the compound identification algorithm, described by Kujawinki and Behn (56) and modified by Minor et al. (57). Briefly, chemical formulas were assigned based on the following criteria: S/N > 6 and mass measurement error < 1 ppm, taking into consideration the presence of C, H, O, N, S, and P and excluding other elements (20). In addition, element combinations were limited to molecular formulas containing ^12^C_0–100_, ^1^H_0–200_, ^16^O_0–30_, ^14^N_0–4_, ^32^S_0–2_, and ^31^P_0–1_ (31). For those peaks when multiple formula candidates were found, we implemented the following rules to further ensure consistent formula assignment: (i) consistently pick the formula with the lowest error and with the lowest number of heteroatoms, and (ii) assignment of one phosphorus atom requires the presence of at least four oxygen atoms (20). We think that formulas retrieved following these criteria were ascribed with a high level of confidence.

Initially, we retrieved 21,253 peaks for our 54 samples (2,029 to 5,456 per sample), within which 9,295 had formula assignments. We found that more than half of the peaks were detected only once across the samples (12,544 in 21,253). To eliminate the interference of those sparse mass spectral peaks, we further refined our raw MS data into a dominant subset, which only contains those peaks that appear more than 6 times for further analysis. (We have six sampling sites in this study.) Finally, we got a dominant MS data set containing 5,254 peaks, with all having formula assignments. The number of peaks in each sample ranged from 1,950 to 4,360. Compounds were plotted on the van Krevelen diagram based on their molar H/C ratio (*y* axis) and molar O/C ratios (*x* axis) (58), which enabled comparisons of the average properties of OM and could be used to assign carbon species to major biochemical classes, which include lipid-, lignin-, protein-, carbohydrate-, tannin-, and condensed aromatic-like compounds (59).

### Estimation of DOM thermodynamic quality.

The Gibbs free energy for the half-reaction of carbon oxidation $\left(\Delta G_{\text{Cox}}^{\circ }\right)$ was calculated for organic compounds to infer their thermodynamic quality (taking into consideration C, H, O, N, P and S, and excluding other elements) (20). $\Delta G_{\text{Cox}}^{\circ }$ is estimated from the empirical equation (26)

$$\Delta G_{\text{Cox}}^{\circ }=60.3-28.5\times \text{NOSC}$$

where NOSC is the nominal oxidation state of carbon, which is estimated by the empirical equation (26)

NOSC  = 4− [(−Z+4C+H−3N−2O+5P−2S)/C]

Here, C, H, N, O, P, and S represent the number of atoms of elements C, H, N, O, P, and S (respectively) in a given organic carbon compound, and Z is the corresponding net charge (we assume a neutral charge per molecule). It is worth noting that the unit of the measurement for $\Delta G_{\text{Cox}}^{\circ }$ is kJ per mol C, rather than per mol substrate. Positive $\Delta G_{\text{Cox}}^{\circ }$ values indicate that the oxidation of carbon must be coupled with the reduction of a terminal electron acceptor (20). Importantly, we expect an organic compound with a higher $\Delta G_{\text{Cox}}^{\circ }$ value to be thermodynamically less favorable at the molecular level (26). The $\Delta G_{\text{Cox}}^{\circ }$ for a given sample in this study was estimated as the median value of all the molecular components within that sample.

### Bacterial 16S rRNA amplicon sequencing and data processing.

Soil total genomic DNA was extracted using the FastDNA Spin kit for soil (MP Biomedicals, Santa Ana, CA). The concentration and quality of the total DNA were determined by spectrophotometry on a Nanodrop ND-2000 and electrophoresis on 1% agarose gel, respectively. The hypervariable V4-V5 region of the bacterial 16S rRNA gene was amplified using primer set 519F/907R and sequenced using an Illumina MiSeq platform (Illumina, Inc., San Diego, CA). Detailed information regarding the PCR procedure can be found in the article by Feng et al. (23). After electrophoresis at 80 V on a 1.5% agarose gel, the PCR products with bright target bands were purified with an Omega Cycle-Pure kit and equimolarly homogenized. Sequencing libraries were generated using the VAHTS Universal DNA Library Prep kit (Vazyme, Nanjing, China). When qualified, the libraries were sequenced on an Illumina MiSeq, and 250-bp paired-end reads were generated.

Amplicon libraries were processed following the DADA2 pipeline to assign the sequences to amplicon sequence variants (ASVs) (60). The primer sequences were removed using cutadapt (61) before starting the dada2 workflow. In brief, the paired-end sequence quality was visualized using the function plotQualityProfile, and then sequences were filtered using function filterAndTrim following recommended parameters combined with quality assessment (i.e., an expected error threshold of 2 combined with maximum ambiguous bases of 0, with forward and reverse sequences truncated to 260 bp and 200 bp, respectively). The filtered reads were then dereplicated to combine identical reads into unique sequences using the function derepFastq. Consensus quality profiles across all the samples were estimated using the function learnErrors. The consensus quality profiles then informed the denoising algorithm, which infers error rates from samples and removed the identified sequencing errors from the samples using the core function dada. All the above analyses were performed using the R package dada2 (v1.12.1) (60). After building the ASV table and removing chimeras, taxonomy was assigned using the Ribosomal Database Project (RDP) classifier (v2.2) (62). A phylogenetic tree was constructed with FastTree using a multiple-sequence alignment made with PyNAST (63). In the end, the original ASV table was rarified to a depth of 7,000 sequences per sample (the fewest in a single sample) to minimize the effects of the sampling effort on the analysis and to allow comparisons between the diversity patterns among the treatments. Considering the potential bias that might exist during the sequence rarefaction, alpha-diversity estimates, including richness and phylogenetic diversity (PD whole tree), were performed on both the rarified and nonrarefied libraries.

### Statistical analysis.

All the statistical analyses were performed using R (R Core Team [https://www.R-project.org/]). Analysis of variance (ANOVA) followed by *post hoc* Tukey’s honestly significant difference (HSD) tests were used to compare the significant differences between molecular categories in the $\Delta G_{\text{Cox}}^{\circ }$ and between sampling sites in the DOM median $\Delta G_{\text{Cox}}^{\circ }$ and the relative abundances of bacterial phylotypes at the class level. Ecosystems perform multiple functions and services (multifunctionality), rather than a single measurable process (32). To quantify this vital provision, we constructed a soil multinutrient cycling (MNC) index by averaging the standardized (Z-score transformation) seven soil nutrient properties: soil organic matter, total nitrogen, alkaline hydrolytic nitrogen, total phosphorus, available phosphorus, total potassium, and available potassium (32). We conducted ordinary least-squares linear regressions between DOM median $\Delta G_{\text{Cox}}^{\circ }$ and important soil properties, such as the bacterial diversity indices, SOM content, soil pH, and soil multifunctionality. Bray-Curtis dissimilarity was used to compute the sparse matrices of the DOM molecules and bacterial communities. The Mantel test and Procrustes rotation (Monte Carlo permutation test; permutation = 999) were conducted to determine whether two distance matrices were significantly correlated. NMDS (nonmetric multidimensional scaling) and envfit were performed to interpret the effects of DOM factors on the bacterial communities. We then conducted Spearman correlations between the DOM median $\Delta G_{\text{Cox}}^{\circ }$ and the abundances of the dominant bacterial phylotypes. In this correlation analysis, we focused on the most ubiquitous bacterial phylotypes (i.e., 1,152 ASVs) that were observed in more than half of samples within each fertilization regimen. The Spearman correlations between the ubiquitous bacterial phylotypes and DOM median $\Delta G_{\text{Cox}}^{\circ }$ as well as SOM content were visualized using the webtool iTOL (Interactive Tree of Life [https://itol.embl.de/]).

### Data availability.

Raw sequences were deposited into the DDBJ database under accession no. DRA006218.
