# Erratum for Zhang et al., “Evaluation of Microbe-Driven Soil Organic Matter Quantity and Quality by Thermodynamic Theory”

**DOI:** 10.1128/mbio.01047-23

**Published:** 2023-05-03

**Authors:** Jianwei Zhang, Youzhi Feng, Meng Wu, Ruirui Chen, Zhongpei Li, Xiangui Lin, Yongguan Zhu, Manuel Delgado-Baquerizo

Volume 12, No. 1, e03252-20, 2021, https://doi.org/10.1128/mBio.03252-20. Page 1, byline: An affiliation designation is missing from the first author’s name. Jianwei Zhang should be affiliated with both State Key Laboratory of Soil and Sustainable Agriculture, Institute of Soil Science, Chinese Academy of Sciences, Nanjing, China, and University of Chinese Academy of Sciences, Beijing, China.

