# Supplementary figures and images for: Evaluation of Microbe-Driven Soil Organic Matter Quantity and Quality by Thermodynamic Theory
Source: mBio. 2021 Feb 23;12(1):e03252-20. doi: 10.1128/mBio.03252-20 (PMC8545108; doi:10.1128/mBio.03252-20)

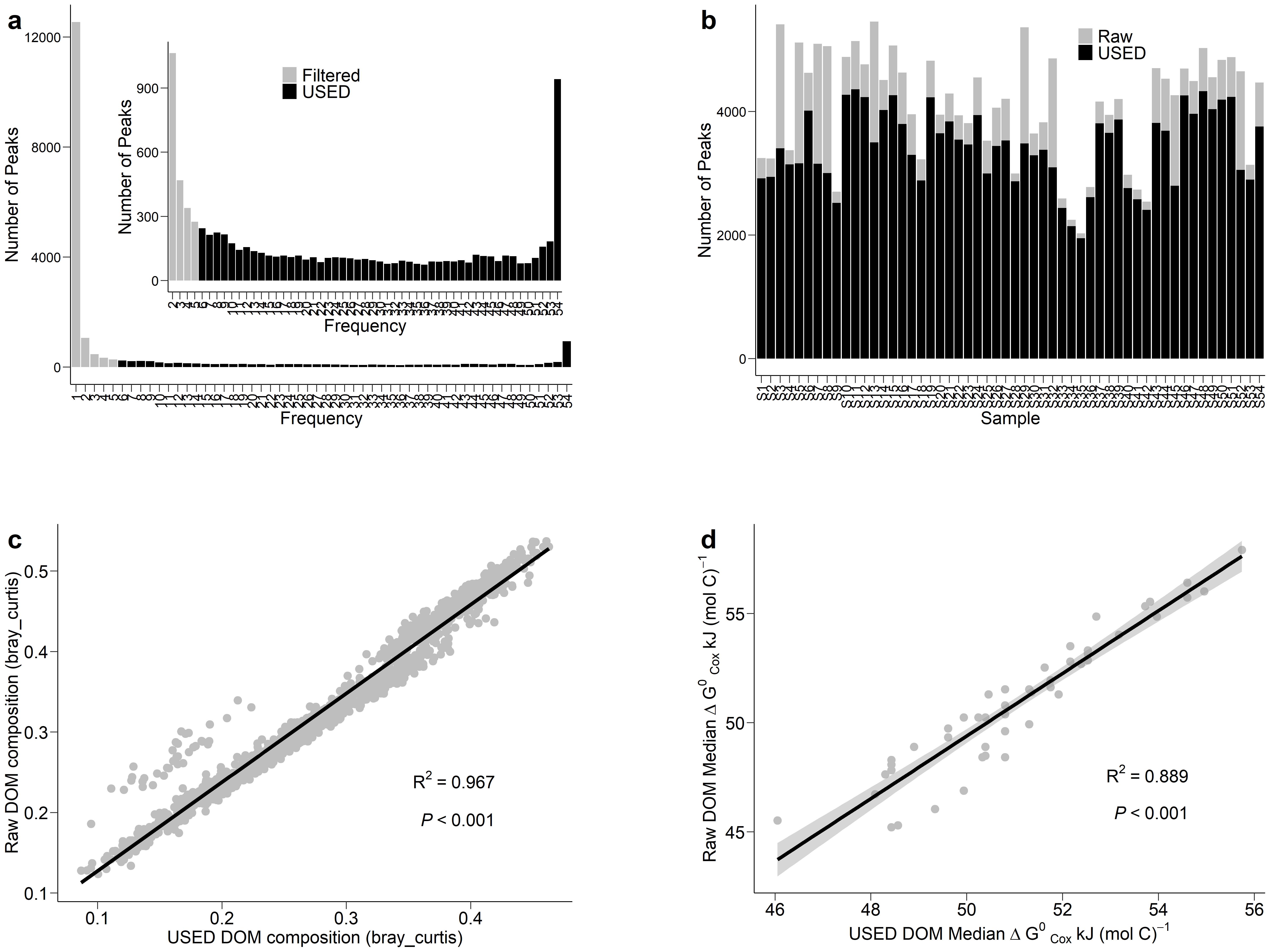

Supplement: FIG S1 [file mbio.03252-20-sf001.jpg]

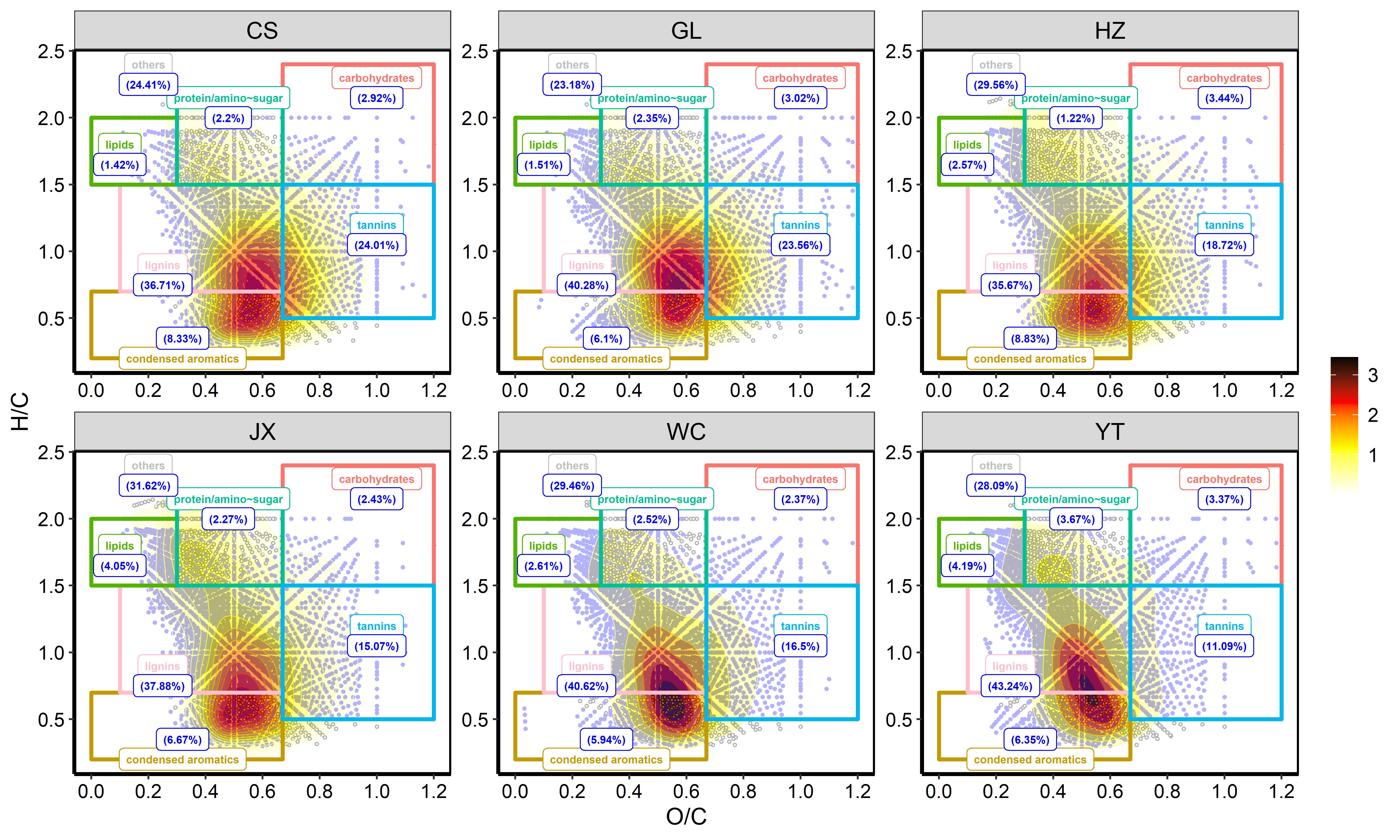

Supplement: FIG S2 [file mbio.03252-20-sf002.jpg]

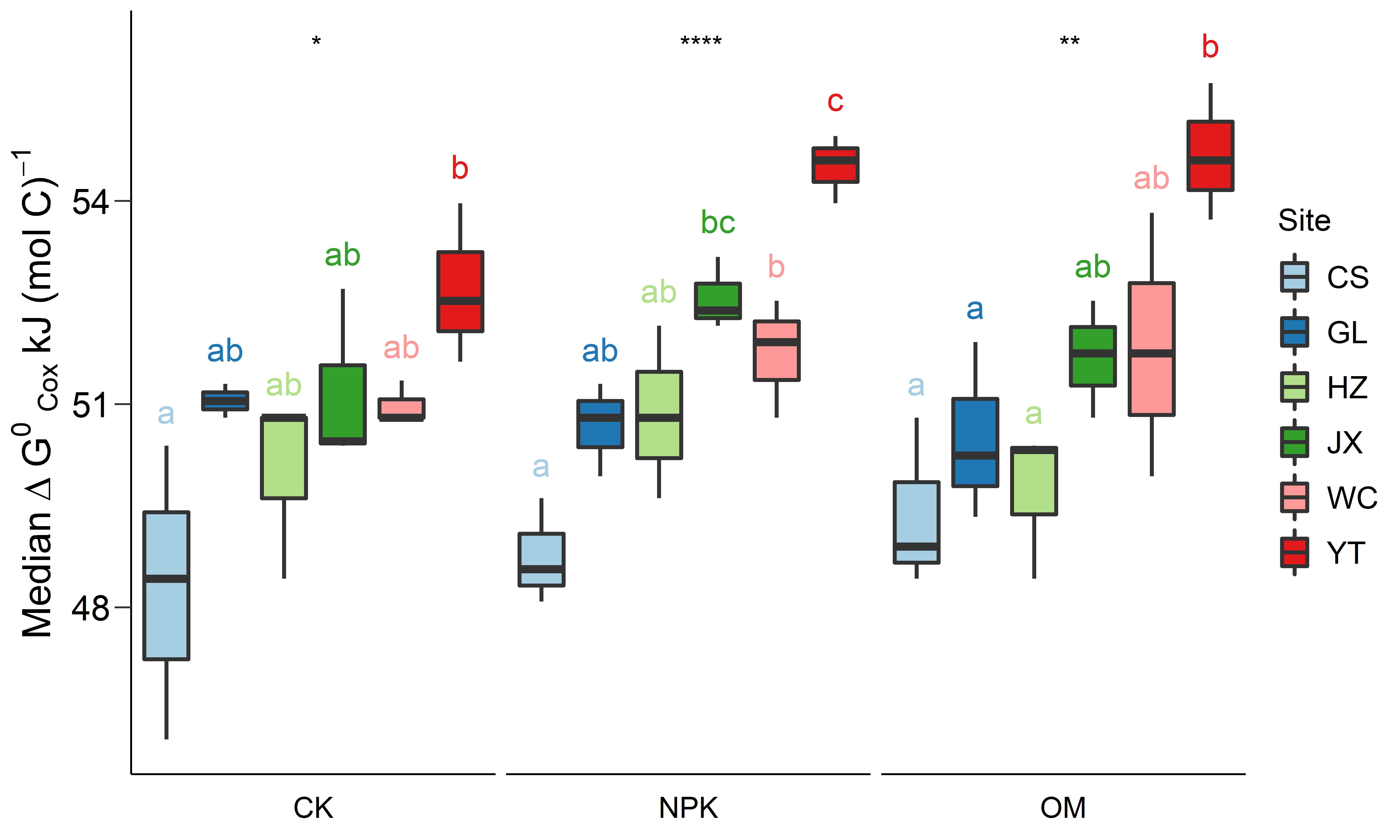

Supplement: FIG S3 [file mbio.03252-20-sf003.jpg]

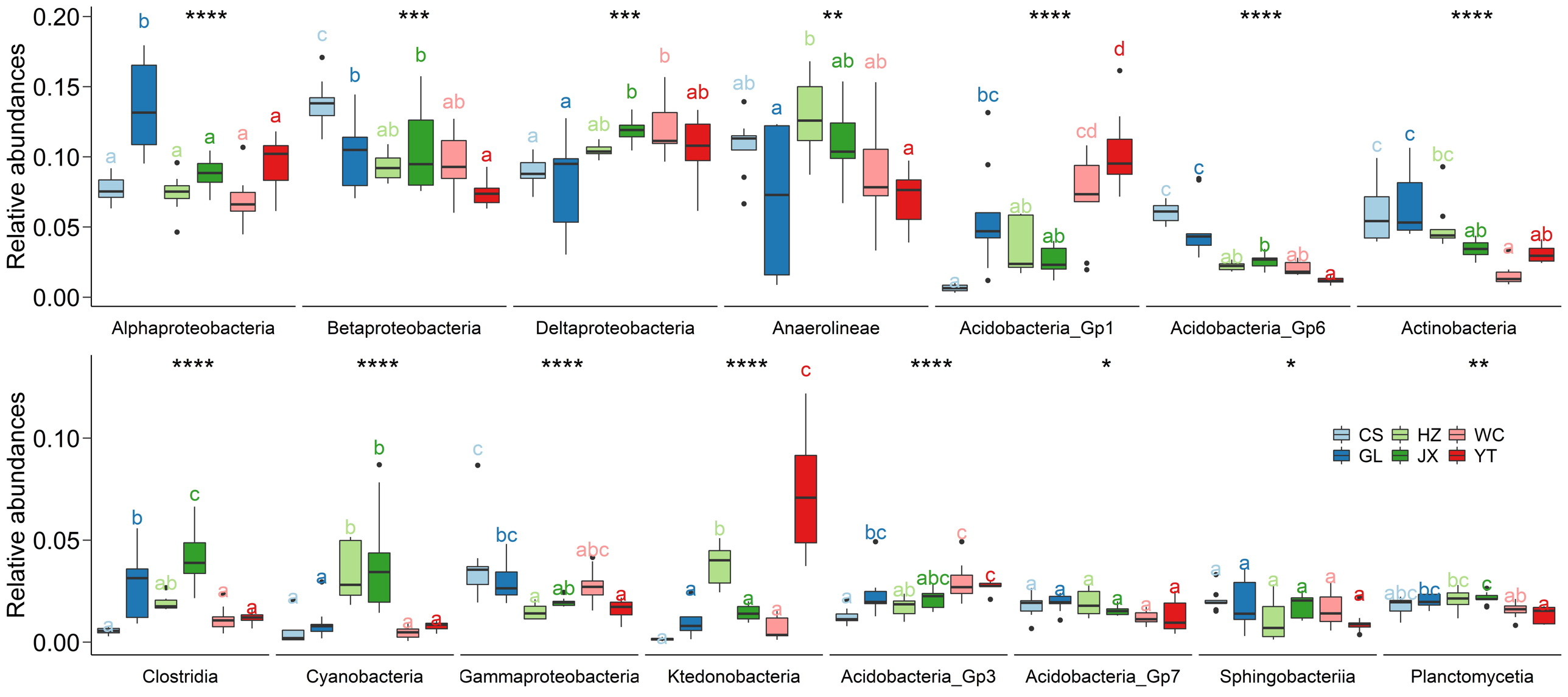

Supplement: FIG S4 [file mbio.03252-20-sf004.jpg]

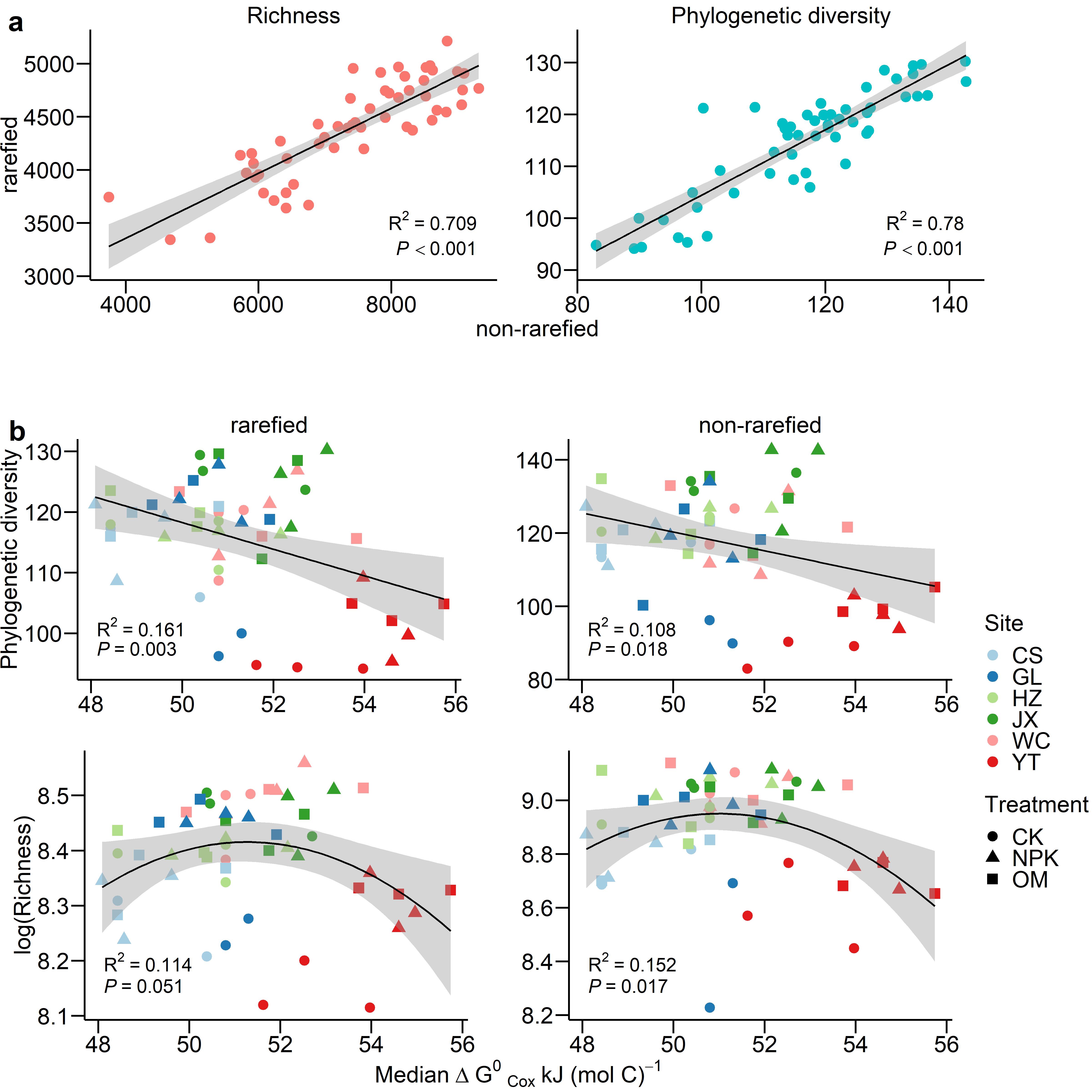

Supplement: FIG S5 [file mbio.03252-20-sf005.jpg]

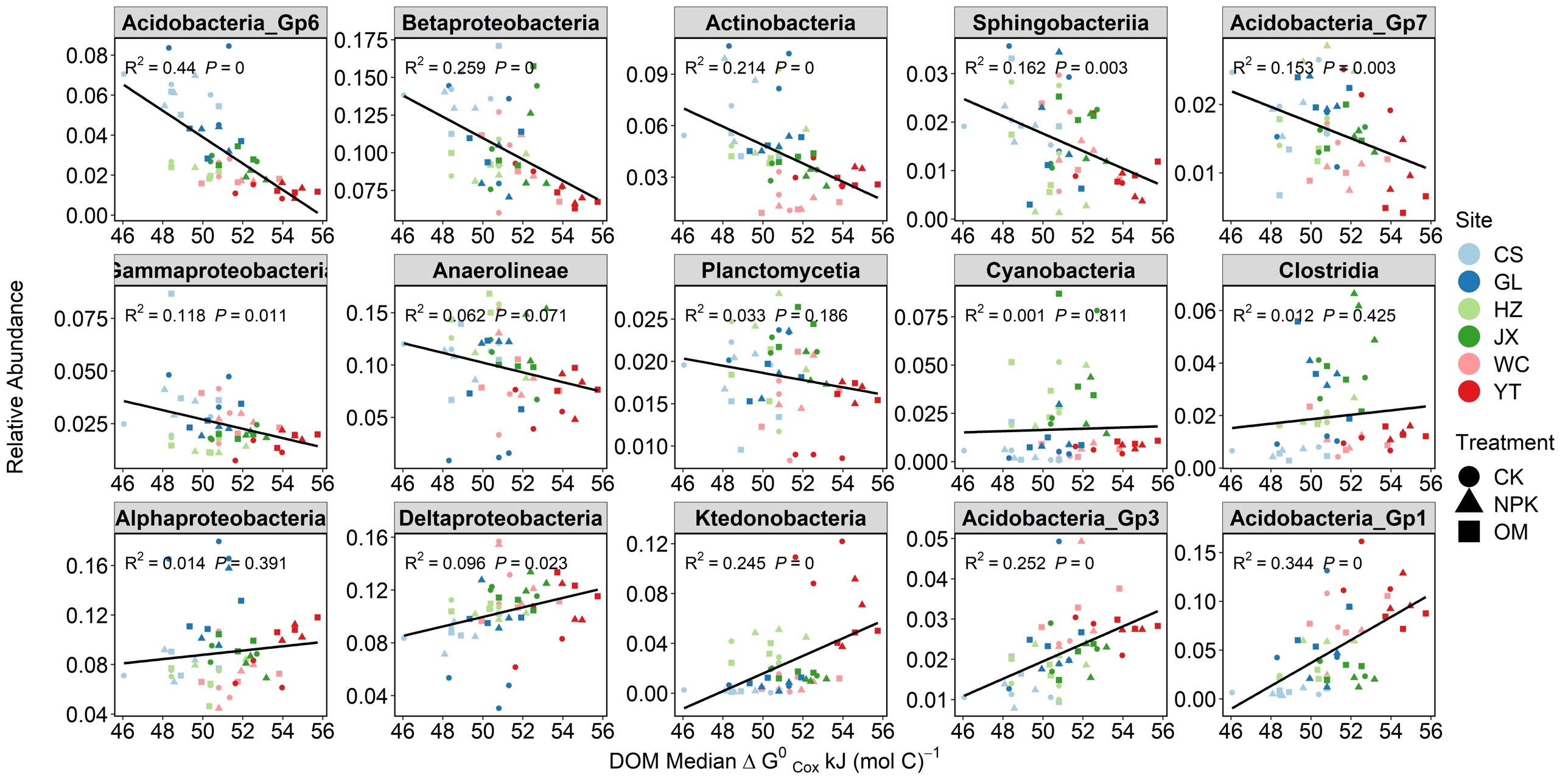

Supplement: FIG S6 [file mbio.03252-20-sf006.jpg]

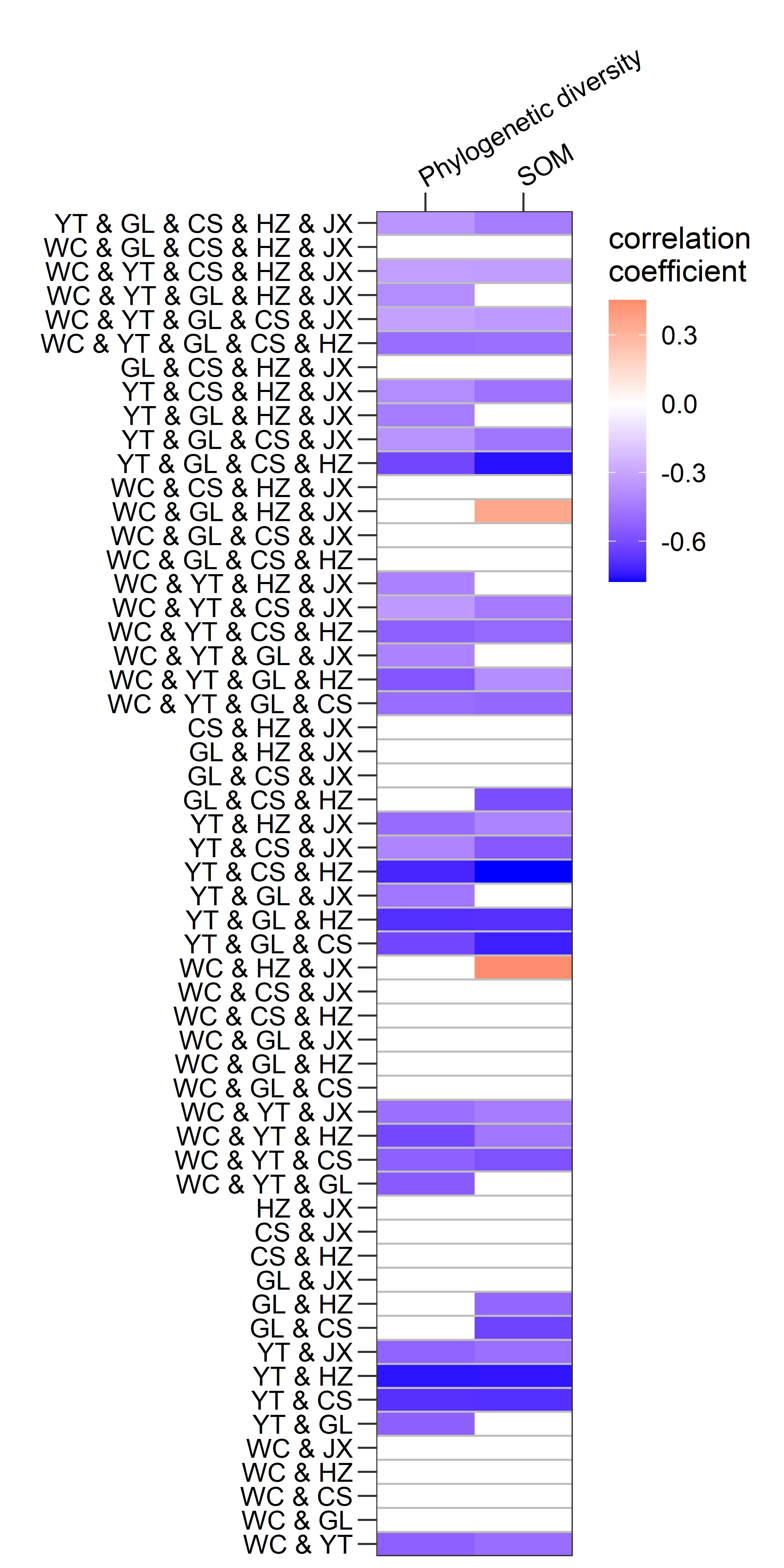

Supplement: FIG S7 [file mbio.03252-20-sf007.jpg]
